# Is It a “Colon Perforation”? A Case Report and Review of the Literature

**DOI:** 10.3389/fmed.2022.817029

**Published:** 2022-03-10

**Authors:** Shuangshuang Lu, Xinyu Yao, Jun Shi, Jian Huang, Shaohua Zhuang, Junfang Ma, Yan Liu, Wei Zhang, Lifei Yu, Ping Zhu, Qiuwei Zhu, Ruxia Shi, Hong Zheng, Dong Shao, Yuyan Pan, Shizhen Bao, Li Qin, Lijie Huang, Wenjia Liu, Jin Huang

**Affiliations:** ^1^The Affiliated Changzhou No.2 People's Hospital of Nanjing Medical University, Changzhou, China; ^2^School of Medicine, Dalian Medical University, Dalian, China

**Keywords:** intrauterine devices, colon perforation, migration, laparoscopic-endoscopic cooperative surgery, hysteroscopy

## Abstract

**Background:**

Intrauterine devices (IUDs) are commonly used as a contraceptive method. IUD migration and colon perforation are rare but serious complications occurring sometimes years after insertion.

**Case:**

A 42-year-old woman with complaints of slight abdominal pain underwent a colonoscopy. Colonoscopy showed that a “nail” had penetrated the ascending colon wall and that an arm of the “nail” was embedded in the colon wall. We did not remove the “nail” rashly under colonoscopy. Considering the safety and effectiveness of the patient's operation, we were able to remove the “nail” easily by performing laparoscopic-endoscopic cooperative surgery (LECS) combined with hysteroscopy at the same time.

**Conclusion:**

We report a case of successful removal of a colonic perforation device by colonoscopy, laparoscopy, and hysteroscopy, which is the first method used.

## Introduction

The intrauterine device (IUD) is one of the most effective measures of contraception available today, with its use increasing yearly. However, they may cause rare but potentially serious complications such as migration through the uterine wall and gastrointestinal perforation (1). Ideal treatment of IUD migration remains controversial (2).

We report a case of ectopic migration of an IUD with perforation of the ascending colon along with a literature review. This case has been reported in line with the SCARE criteria (3).

## Case Report

**Figure 1:**
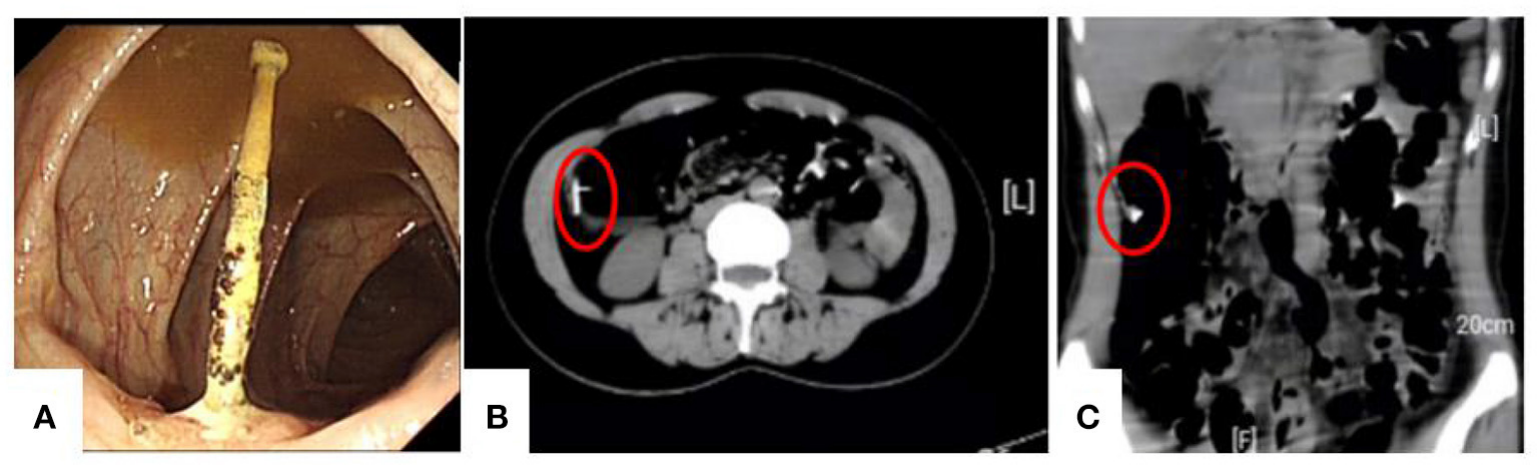
Colonoscopy and abdominal CT findings. Colonoscopy showed a foreign body similar to a nail in the ascending colon, and a local ulcer was formed **(A)**. CT showed a foreign body through the wall of the colon **(B,C)**.

A 42-year-old female presented in our hospital with slight abdominal pain. On admission, the patient's vital signs were normal. In abdominal examination, tenderness without rebound tenderness was felt in the lower abdomen. She had no previous disease history, and her fertility history was 3-0-0-3. We arranged a colonoscopy for the patient, and intestinal preparation was carried out according to the 2015 ASGE guidelines. The whole intestinal preparation process is smooth. During the colonoscopy examination, a small nail was observed protruding through the intestinal wall (Figure 1A). It was firmly adherent to the colon wall. To avoid causing any damage to the intestine, we decided to discontinue the examination to confirm the source of the nail. The rest of the

colonoscopy was eventless. Abdominal computed tomography (CT) was performed and showed a foreign body through the wall of the colon (Figure 1). We inquired about the patient's medical history in detail. The patient denied a history of foreign body swallowing. She underwent IUD implantation 6 months after her first delivery 18 years ago. Surprisingly, 2 years after IUD placement, she became pregnant and gave birth smoothly. At that time, uterine ultrasound did not show the IUD ring, so it was thought to have fallen out naturally. Then, the patient conceived naturally again and gave birth. Considering this reproductive history, we believe that the nail in the intestinal cavity is likely to be the IUD.

**Figure 2:**
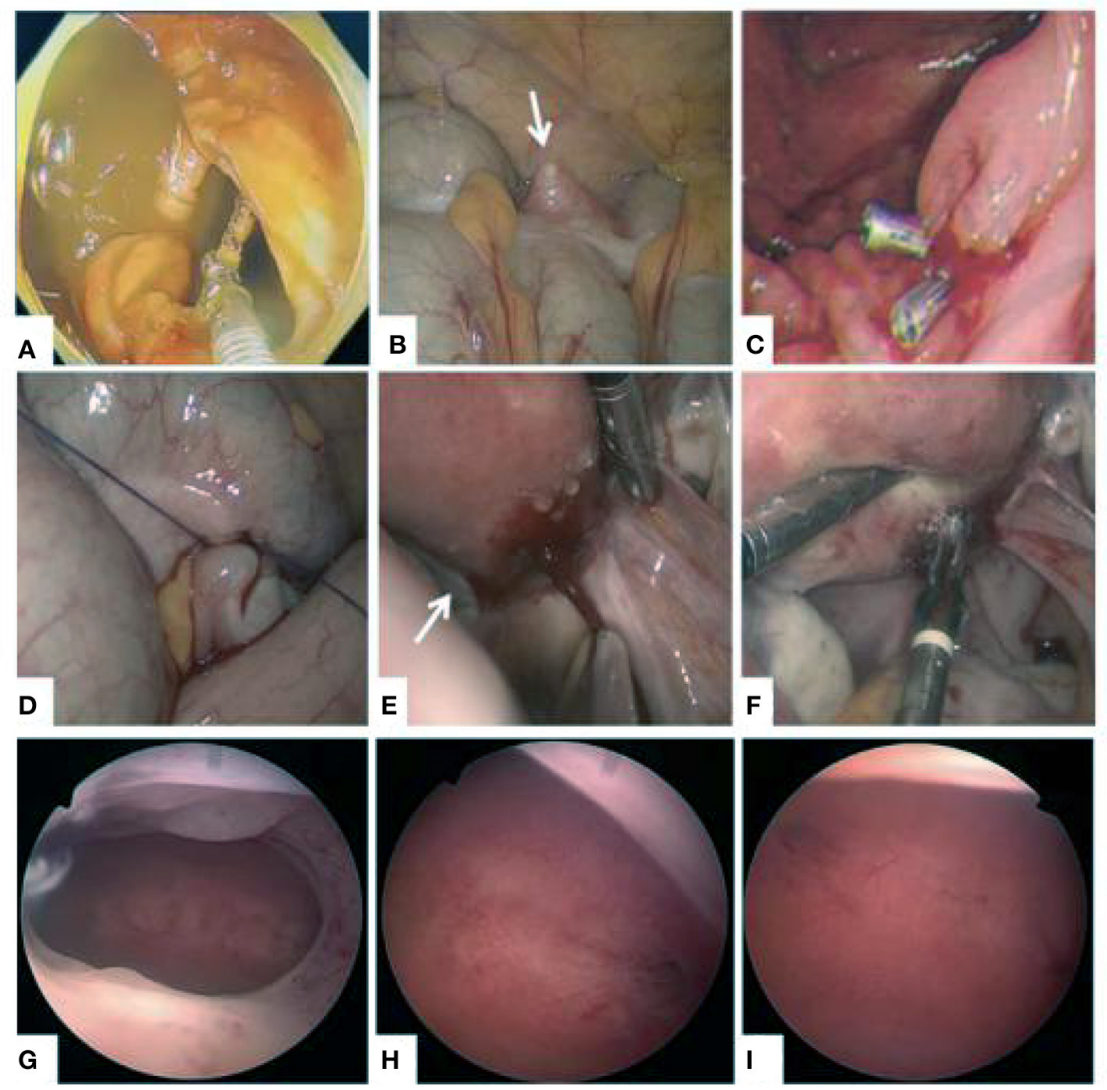
Laparoscopic-endoscopic cooperative surgery and no obvious abnormality were found during hysteroscopy. **(A)** The IUD was removed under laparoscope. **(B)** The IUD embedded in the colon wall showed a white protuberance outside the cavity (arrow). **(C)** Haemoclips at the wound site to prevent perforation of the colon. **(D)** The weak intestinal wall was sutured to prevent perforation. **(E)** Localized erosion at the posterior wall of the uterus and dense adhesions between the uterus and colon. **(F)** Bipolar coagulation to stop bleeding. **(G)** Morphology of uterine cavity. **(H)** Opening of right fallopian tube. **(I)** Opening of left fallopian tube.

**Figure 3:**
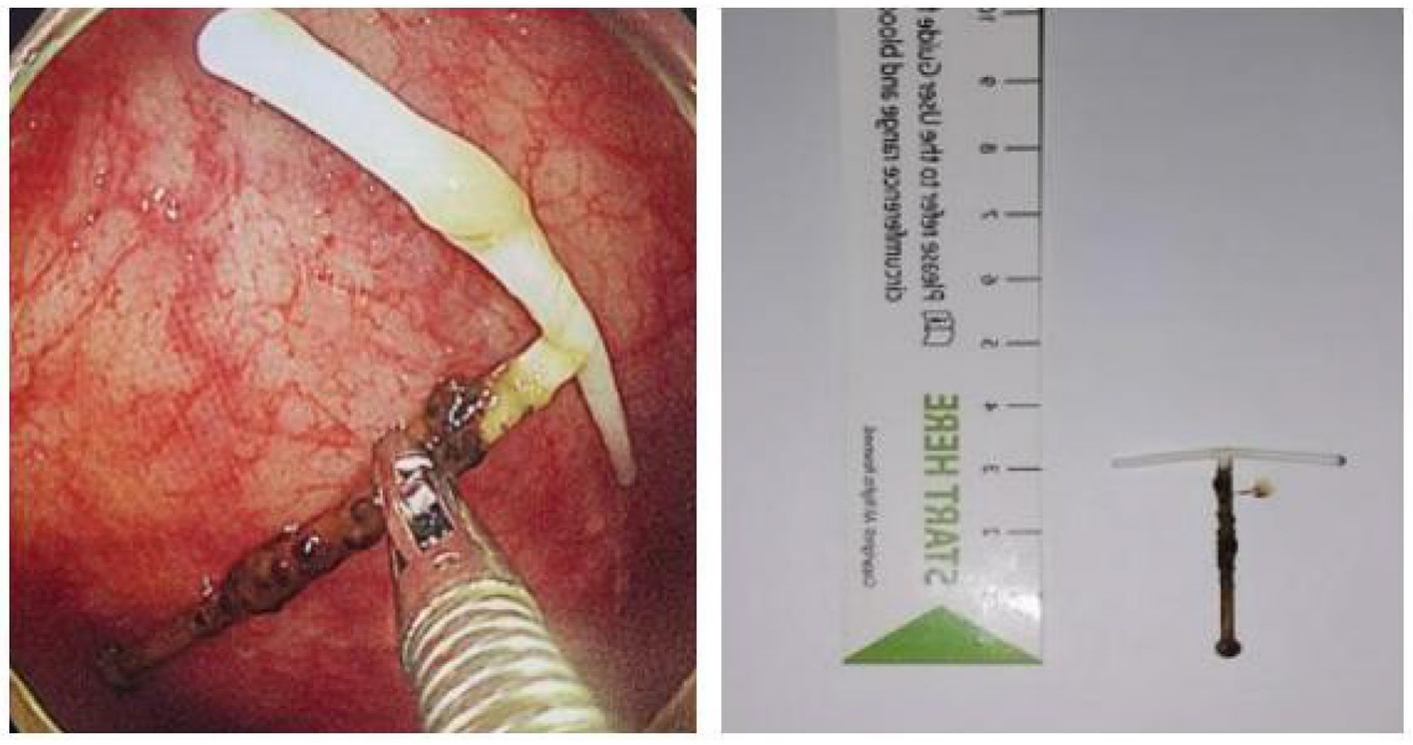
The IUD was removed successfully.

To ensure the safety and effectiveness of the operation, we performed laparoscopic-endoscopic cooperative surgery (LECS) and hysteroscopy at the same time. During the operation (Figure 2), granulomas formed in the anterior wall of the ascending colon, protruding on the surface. A nail-like foreign body was removed from the intestine by foreign body forceps under enteroscopy. A clip was used for hemostasis and closure of wounds. As seen by laparoscopy during the operation, the wounds on the lateral intestine were eroded after removal of the IUD. Silk thread was used for preventive sutures at the weak part of the intestinal wall to prevent secondary perforation. Three laparoscopic ports were used. The intraoperative blood loss was minimal. Examination of the posterior wall of the uterus showed hyperemia and erosion, with a diameter of 1.5 cm, surrounding granulation tissue. No obvious abnormality was found during hysteroscopy (Figure 2; Supplementary Video 1). The operation (Figure 3) was carried out smoothly, and there was no special discomfort after the operation. She was discharged after 5 days of postoperative recovery. One month after the operation, the patient went to the clinic for follow-up and a re-check abdominal CT, and no abnormality was found.

## Discussion

In this case report, the patient underwent IUD placement after initial production but did not go to the gynecological clinic regularly thereafter. In the case of accidental pregnancy, an IUD was not found in the uterine cavity by ultrasonography, and it was mistakenly considered that the IUD had fallen out of the vagina and was excreted from the body. After the patient's second delivery, the contraceptive method used was safe period contraception, and the effect of this method was not good. Later, the patient became pregnant again and gave birth for a third time.

A literature search was conducted in PubMed on the 1st of August 2021 using the search terms “IUD perforation” and “colon”, covering 1969 to the present. There were 38 matches in total. After removing non-colon and incomplete data-related issues, 26 articles were included from the literature (1, 4–28). We reviewed 31 cases of IUD-related uterine and subsequent intestinal penetrations.

In most cases (Table 1), the patient placed an IUD during lactation after the initial delivery. At that time, the uterus was soft, and it was likely that a rare complication of placing an IUD occurred, which was that it was freed from the uterus and penetrated into the intestinal cavity, resulting in sterile perforation. The IUD is one of the most effective, safe, and economical contraceptive methods (4). However, IUDs have been associated with serious complications such as bleeding, uterine perforation, and bowel perforation (29). Perforation usually occurs immediately after insertion. However, it can occur years later. A possible mechanism for colonic penetration is adherence of the copper IUD to the pericolonic fat, followed by local inflammation and eventual penetration into the colon (5). Another less likely mechanism is uterine enlargement during the patient's pregnancy, physically displacing the IUD into the colon (30).

**Table 1:** Demographics of patients in the included case series.

	**Maximum**	**Minimum**	**Average^**a**^**
Age, years	77	20	36.3 ± 11.1
Time from IUD insertion to finding it in the intestine	35 years	2 weeks	6.47 ± 7.67

a *Mean ± standard deviation, years*.

When the literature was analyzed (Table 2), most IUDs were perforated in the sigmoid colon. Chronic abdominal pain was the main manifestation of IUD migration, and acute abdominal pain was another main manifestation. Most patients could have the IUD removed by laparoscopy. In most cases, adhesions and bowel perforation were thought to have led to the abandonment of attempts at laparoscopy and subsequent laparotomy. Laparoscopy combined with hysteroscopy was used to remove the IUD in 2 cases (7, 26), which was related to the direction and shape of the IUD insertion. Two cases had the IUD removed without operation (9, 11). One patient did not receive treatment because she was older and had no clinical symptoms (9), and another died soon after due to other malignancies (11). In one case, there was imperfect removal because the 1-cm right arm of the IUD was suspected to have been left in the lumen of the colon (12). More rarely, there was a case (9) in which two different IUDs were found penetrating the colorectal wall. Although it is a general recommendation to remove all migrated uterine devices to avoid complications (8), leaving the device in place in asymptomatic patients should also be considered.

**Table 2:** Clinical presentation and intraoperative findings of intracolon IUDs.

**Catalog**	**Number of patients**	**Total (%)**
**Symptom** ^**a**^		
Abdominal pain	23	74.2
Vaginal bleeding	2	6.5
Irregular menstruation	3	9.7
Backache	2	6.5
Bloody stool	2	6.5
Acute abdomen	1	3.2
Perianal pain	1	3.2
Difficult sexual intercourse	3	9.7
Asymptomatic	5	16.1
**Location of misplaced IUD** ^**b**^		
Sigmoid colon	24	77.4
Ileocecal part	3	9.7
Ascending colon	2	6.5
Hepatic flexure of colon	1	3.2
Transverse colon	1	3.2
Splenic flexure of colon	1	3.2
Descending colon	2	6.5
Rectum and rectosigmoid junction	3	9.7
**Treatment** ^**c**^		
Laparoscope	14	45.2
Laparotomy	13	41.9
Colonoscopy	1	3.2
Hysteroscopy	2	6.5
Untreated	2	6.5
**Total** ^**d**^	31	

a *Reviewing 31 cases in the literature, their symptoms were clearly recorded. Abdominal pain is often accompanied by other symptoms, such as fever, diarrhea, back pain, difficulty in sexual intercourse, etc*.

b *A rare elderly woman who had two IUDs placed was found to have intestinal displacement at the same time, located in the transverse colon and rectum*.

c *IUDs were removed by hysteroscopy in 2 cases, 1 case combined with laparoscopy, and the other case was not explained*.

d *Number of cases in which the patient's research data were complete*.

We report a case of IUD perforation found in the ascending colon that was successfully removed by colonoscopy, laparoscopy, and hysteroscopy. This operation is minimally invasive, safe, and effective. Injury to the intestine, abdominal cavity and uterine cavity were evaluated at the same time. This case was analyzed retrospectively. It was suggested that the patient should have regular re-examination 6 weeks after IUD placement (4). When the IUD is found to disappear in the uterine cavity, it should not be excluded that there is the possibility of ectopic migration, and physicians should be vigilant for the potential for intestinal perforation. Because there is a risk of perforation when removing intestinal foreign bodies under endoscopy, laparoscopy can be used to observe the abdominal side of the intestinal wall when removing intestinal foreign bodies under endoscopy to improve the safety and success rate of the operation.

The diagnosis of and operation for IUD perforations are complicated and difficult and may require the combined operation of digestive physicians, gastrointestinal surgeons, and gynecologists. However, due to the extensive use of IUDs (25), the diagnosis and treatment of this issue cannot be ignored. The combination of laparoscopy and colonoscopy, and even hysteroscopy, may increase the economic burden on patients, but it is helpful to identify the location, degree, and scope of the lesions. IUDs can be taken out accurately to reduce unnecessary damage to achieve minimal invasiveness and reduce the role of recurrence. In addition, the treatment can also identify uterine cavity injury to increase the probability of pregnancy and improve gastrointestinal tract healing and quality of life. It is an effective method in clinical practice. However, due to the complexity and intersection of surgery and specialty, it is suggested that it should be popularized gradually under the condition of fully evaluating the patient's condition and coordinating with relevant specialists.

## Conclusion

We report a case of successful removal of a colonic perforation device by colonoscopy, laparoscopy, and hysteroscopy, which is the first method used. With the popularity of minimally invasive concepts and the continuous development of minimally invasive technology, multimirror combined technology will have high application value in the treatment of intestinal foreign body removal.

## Author Contributions

YL found intestinal foreign bodies in the renamed patient during the initial colonoscopy. WL, JinH, XY, JiaH, SL, WZ, LY, SZ, and JM have done a lot of work in colonoscopy, treatment, and postoperative follow-up. JS, PZ, and QZ participated in the laparoscopic operation. RS and HZ performed hysteroscopy for the patient. SB, LH, and LQ made contributions to intraoperative cooperation and postoperative nursing. DS performed intraoperative anesthesia for the patient. YP gave guidance on the treatment and medication of patients. SL sorted out all the materials and wrote a paper with XY and JS. JinH and WL finally revised the manuscript. The manuscript was written through the contributions of all authors. All authors have given approval to the final version of the manuscript.

## Conflict of Interest

The authors declare that the research was conducted in the absence of any commercial or financial relationships that could be construed as a potential conflict of interest.

## Publisher's Note

All claims expressed in this article are solely those of the authors and do not necessarily represent those of their affiliated organizations, or those of the publisher, the editors and the reviewers. Any product that may be evaluated in this article, or claim that may be made by its manufacturer, is not guaranteed or endorsed by the publisher.

## Supplementary Material

Supplementary Video 1 Hysteroscopy went well.
